# Range-separated hybrid functionals for accurate prediction of band gaps of extended systems

**DOI:** 10.1038/s41524-023-01064-x

**Published:** 2023-06-21

**Authors:** Jing Yang, Stefano Falletta, Alfredo Pasquarello

**Affiliations:** Chaire de Simulation à l’Echelle Atomique (CSEA), Ecole Polytechnique Fédérale de Lausanne (EPFL), Lausanne, CH-1015 Switzerland

**Keywords:** Computational methods, Electronic structure, Electronic properties and materials

## Abstract

In this work, we systematically evaluate the accuracy in band gap prediction of range-separated hybrid functionals on a large set of semiconducting and insulating materials and carry out comparisons with the performance of their global counterparts. We observe that all the range-separated hybrid functionals that correctly describe the long-range dielectric screening significantly improve upon standard hybrid functionals such as PBE0 and HSE06. The choice of the short-range Fock exchange fraction and the screening length can further reduce the predicted error. We then propose a universal expression for the selection of the inverse screening parameter as a function of the short-range and long-range Fock exchange fractions, which results in a mean absolute error as small as 0.15 eV for band gap prediction.

## Introduction

Accurate prediction of materials band gaps is key to studying the electronic and optical properties of semiconductors and insulators. However, Kohn–Sham density functional theory (KS-DFT)^1,2^ intrinsically underestimates the fundamental band gap *E*_g_, defined as the difference between the ionization potential and the electron affinity^3^. Even in exact KS-DFT, the predicted band gap deviates from the fundamental band gap by Δ_xc_, the discontinuity in the exchange-correlation potential^4^. In the local-density approximation or in the generalized gradient approximation, this leads to the underestimation of the band gaps by about 50%^5,6^. More recent developments in semilocal functionals, such as the modified Becke–Johnson potential^7^, GLLB-SC^8^, and TASK^9^, show improvement in band gap predictions. However, to date, an average error of 0.5 eV persists when applying the best-performing semilocal functionals^10,11^.

The generalized KS theory resolves the band gap prediction problem by admixing a fraction of Fock exchange with the semilocal DFT exchange, thus opening up the band gap^12^. For example, the global hybrid-functional PBE0 includes a fraction *α* = 0.25 of Fock exchange, which was found to optimize the atomization energies of molecules^13–15^. Heyd et al. developed the screened hybrid-functional HSE06^16,17^, which has a mixing parameter of 0.25 in the short range and reproduces semilocal exchange in the long range. In spite of their widespread use, the hybrid functionals adopting fixed mixing parameters, such as PBE0 and HSE06, are not universally applicable. For solid-state systems, these functionals perform best on materials with intermediate band gaps^18^. However, their accuracy greatly deteriorates for wide band gap materials, such as MgO and LiF, and narrow band gap materials, such as Si and Ge^19,20^. This inadequacy led to the development of nonempirical hybrid functionals. In these functionals, the mixing parameters are determined nonempirically by enforcing certain exact constraints on the exchange-correlation potential^4^. Through adopting material-specific fractions of Fock exchange, the nonempirical hybrid functionals are promising in achieving more uniform accuracy in band gap prediction, as well as in predicting other electronic, optical, and structural properties of semiconductors and insulators^21–25^.

Based on the specific exact constraints imposed, nonempirical hybrid functionals are sought according to two lines of thought. The first group, often denoted dielectric-dependent (DD) hybrid functionals, is built by connecting *α* with the macroscopic dielectric constant *ϵ*_*∞*_^19,26^. The simplest form of this group, DD-PBE0, admixes a fraction *α* = 1/*ϵ*_*∞*_ of Fock exchange. These functionals correctly describe the long-range interaction, which asymptotically approaches $-1/({\epsilon }_{\infty }| {{{\bf{r}}}}-{{{{\bf{r}}}}}^{{\prime} }| )$^27^. DD-PBE0 allows for strong screening in the case of narrow band gap materials and weak screening in the case of wide band gap materials, and thus greatly enhances the uniformity of the achieved accuracy. This idea has subsequently been combined with the development of range-separated hybrid (RSH) functionals, in which different Fock fractions are admixed in the long range and in the short range, separated through the use of an inverse screening length *μ*^28–30^. Hence, DD-RSH functionals generally adopt a long-range Fock fraction *α*_*l*_ = 1/*ϵ*_*∞*_ and various differing strategies for determining the short-range Fock fraction *α*_*s*_ and the inverse screening length *μ*^20,31,32^.

The second group of nonempirical hybrid functionals is constructed by imposing the piecewise linearity condition, which asserts that the ground-state energy *E*(*N*) as a function of electron number *N* must be linear upon electron occupation between integer electron numbers^33^. Through Janak’s theorem^34^, this constraint translates to the single-particle energy level of the highest occupied state being constant irrespective of its occupation, a constraint known as the generalized Koopmans’ condition. To construct a piecewise linear hybrid functional, the mixing parameters can be found by enforcing Koopmans’ condition on a localized electronic state. These functionals were first applied to organic molecules^35,36^ and more recently to extended systems^22,24,37–45^. They were demonstrated to be especially useful for materials with heterogeneous dielectric screening, for example, for two-dimensional materials^21,46^ and interfaces^47^.

Despite these recent developments of nonempirical hybrid functionals, the methods generally adopt different ways of choosing *α* and *μ* values and there has been a lack of systematic comparison among these choices. The average errors in band gap predictions are reported on different sets of materials, with different material structures, pseudopotentials, or convergence parameters. Furthermore, some of these functionals require a rather cost-intensive construction process, hindering their widespread use^20,23,37,38,41,43^. In this work, we present a comprehensive comparison of the performance of six nonempirical hybrid functionals by evaluating their accuracy in predicting the band gaps for a variety of semiconducting and insulating materials. We show that nonempirical hybrid functionals significantly outperform standard hybrid functionals such as PBE0 and HSE06. We then provide a detailed analysis on how the fraction of Fock exchange and the inverse screening parameter affect the predicted band gaps. We show that available methods for determining the inverse screening parameter do not lead to an improvement in the overall accuracy compared to adopting a fixed value. In light of this observation, we further propose an analytical expression for setting the inverse screening parameter as a function of the fractions of Fock exchange in the short and long range. The optimal functional constructed in this way further reduces the average error in the band gap prediction to 0.15 eV.

## Results and discussion

Table 1 gives a summary of the hybrid functionals considered in this work. We start with global hybrid functionals in which the fraction of Fock exchange is defined by a single parameter *α*. In the commonly used PBE0 functional, *α* is set to 0.25^14^. In DD-PBE0, *α* is set to 1/*ϵ*_*∞*_.

**Table 1:** List of hybrid functionals considered in this work with their corresponding mixing parameters

		*α*_*s*_	*α*_*l*_	*μ*
global	PBE0	0.25	0.25	–
	DD-PBE0	1/*ϵ*_*∞*_	1/*ϵ*_*∞*_	–
	K-PBE0	*α*_K_^43^	*α*_K_	–
*α*_*s*_ = 0.25	HSE06	0.25	0	0.106 bohr^−1^
	TF	0.25	1/*ϵ*_*∞*_	*μ*_TF_^31^
	${\mu }_{{{{\rm{fix}}}}}^{{\alpha }_{s} = 0.25}$	0.25	1/*ϵ*_*∞*_	0.71 bohr^−1^
*α*_*s*_ = 1	DSH	1	1/*ϵ*_*∞*_	*μ*_DSH_^32^
	DD-CAM	1	1/*ϵ*_*∞*_	*μ*_DD-CAM_^20^
	${\mu }_{{{{\rm{fix}}}}}^{{\alpha }_{s} = 1}$	1	1/*ϵ*_*∞*_	0.71 bohr^−1^
	K-CAM-*α*_*s*_	*α*_*s*,K_^43^	1/*ϵ*_*∞*_	0.106 bohr^−1^

The specific values of the mixing parameters for each material can be found in the Supplementary Information.

RSH functionals adopting the Coulomb attenuating method (CAM)^48^ separate the nonlocal exchange potential into short-range and long-range parts through an error function with inverse screening length *μ*:

1 $$\frac{1}{|{\mathbf{r}}-{\mathbf{r}}^{\prime}|}=\underbrace{\frac{1-{\mathrm{erf}}(\mu|{\mathbf{r}}-{\mathbf{r}}^{\prime}|)}{|{\mathbf{r}}-{\mathbf{r}}^{\prime}|}}_{\text{SR}}+\underbrace{\frac{{\mathrm{erf}}(\mu|{\mathbf{r}}-{\mathbf{r}}^{\prime}|)}{|{\mathbf{r}}-{\mathbf{r}}^{\prime}|}}_{\text{LR}}.$$

In this way, the exchange potential is defined as follows:

2 $$\begin{array}{lll}{v}_{x}({{{\bf{r}}}},{{{{\bf{r}}}}}^{{\prime} })&=&{\alpha }_{s}{v}_{x}^{{{{\rm{SR-Fock}}}}}({{{\bf{r}}}},{{{{\bf{r}}}}}^{{\prime} };\mu )+(1-{\alpha }_{s}){v}_{x}^{{{{\rm{SR-PBE}}}}}({{{\bf{r}}}};\mu )\\ &+&{\alpha }_{l}{v}_{x}^{{{{\rm{LR-Fock}}}}}({{{\bf{r}}}},{{{{\bf{r}}}}}^{{\prime} };\mu )+(1-{\alpha }_{l}){v}_{x}^{{{{\rm{LR-PBE}}}}}({{{\bf{r}}}};\mu ),\end{array}$$

where ${v}_{x}^{{{{\rm{PBE}}}}}$ and ${v}_{x}^{{{{\rm{Fock}}}}}$ are the semilocal and the nonlocal exchange potentials, respectively, with their short-range and long-range component fractions specified by *α*_*s*_ and *α*_*l*_. The parameter *α*_*l*_ is generally set to 1/*ϵ*_*∞*_ to comply with the exact condition of the asymptotic potential in the long range, as done in DD functionals^4,27^. Depending on how *α*_*s*_ and *μ* are chosen, various versions of RSH functionals can be constructed. Here, we group them into two main classes based on the choice of *α*_*s*_. In the first class, *α*_*s*_ is set to 0.25, like in PBE0. The widely used hybrid-functional HSE06 belongs to this class, with *α*_*l*_ set to 0 and *μ* to 0.106 bohr^−1 ^^16,17^. Another common choice of *μ* is the Thomas-Fermi (TF) screening parameter^31,49^, which is defined as follows:

3 $${\mu }_{{{{\rm{TF}}}}}={\left(\frac{3n}{\pi }\right)}^{\frac{1}{6}},$$

where *n* is the valence electron density. Here, all the electrons in the outer shell are counted as valence electrons^32,50^. For example, we take two valence electrons for Ca and thirteen valence electrons for Ga.

In the second class, *α*_*s*_ is set to 1. Two recently proposed functionals belong to this class: the DD-CAM^20^ and the doubly screened hybrid (DSH) functional^32^. The two functionals use the same settings for *α*_*s*_ and *α*_*l*_, but adopt different settings for *μ*. In the former, *μ* is nonempirically determined through fitting the dielectric function calculated from linear response^20^. In the latter, *μ* is defined by the analytical expression:

4 $${\mu }_{{{{\rm{DSH}}}}}=\frac{4}{3}{\left[\frac{1}{\gamma }\left(\frac{1}{{\epsilon }_{\infty }}+1\right){\mu }_{{{{\rm{TF}}}}}^{2}\right]}^{\frac{1}{2}},$$

in which *γ* is empirically set to 1.563. To determine how the material-specific values of *μ* influence the overall accuracy of band gap predictions, we also consider setting *μ* to a fixed value of 0.71 bohr^−1^ for both classes with *α*_*s*_ = 0.25 and *α*_*s*_ = 1 (${\mu }_{{{{\rm{fix}}}}}^{{\alpha }_{s} = 0.25}$ and ${\mu }_{{{{\rm{fix}}}}}^{{\alpha }_{s} = 1}$). This value for the inverse screening parameter has been determined in ref. ^20^ from an average over a large variety of materials.

We also include in our comparison two functionals satisfying the piecewise linearity condition, K-PBE0 and K-CAM^36,37,51^. The K-PBE0 functional is a global one, for which the mixing parameter *α* is determined by inserting an atomic probe into the material system^38,43^. One then systematically varies *α* until the localized electronic state of the probe is constant irrespective of its occupation. Thus, the value *α* = *α*_K_ found in this way satisfies the piecewise linearity condition. The K-CAM functional is range-separated with *α*_*l*_ = 1/*ϵ*_*∞*_ and *μ* = 0.106 bohr^−1^ as in HSE06. The short-range mixing parameter *α*_*s*_ is determined by enforcing the piecewise linearity condition on a localized potential probe, in the same way as for K-PBE0.

In the following sections, we give a detailed analysis of how the functional forms with their mixing parameters influence the achieved accuracy in predicting band gaps. Specifically, we focus on the dependence on *α* for global hybrid functionals, and on the combined dependence on *α*_*s*_ and *μ* for RSH functionals. Following this analysis, we propose a universal formulation for choosing the inverse screening parameter *μ* as a function of *α*_*s*_ and *α*_*l*_. Last, we give a comprehensive comparison of the various functionals in terms of their accuracy and discuss strategies for optimizing RSH functionals.

### Global hybrid functionals

**Fig. 1:**
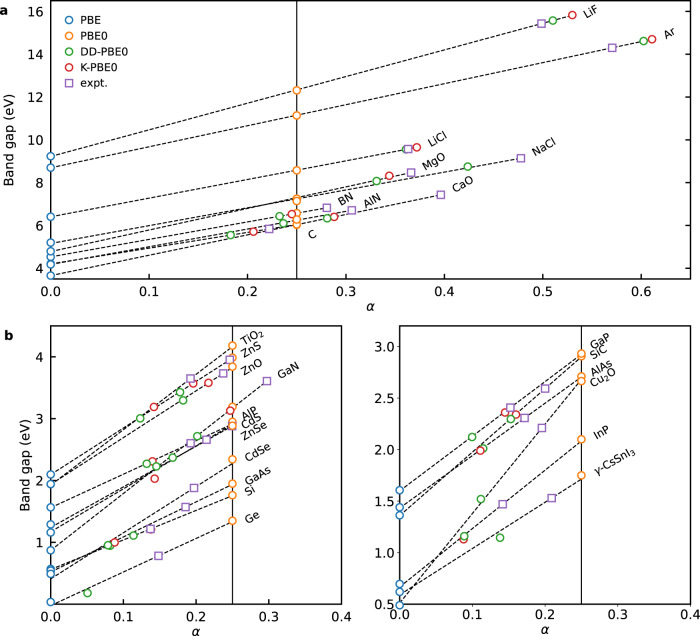
Band gap vs. *α*. Band gaps calculated with global hybrid functionals as a function of *α* in comparison with experimental values for materials **a** with experimental band gaps larger than 5 eV and **b** with experimental band gaps smaller than 5 eV (plotted in two separate panels for clarity). Data points for the same material are fitted to a linear relationship as indicated by the dashed lines. The experimental band gaps are then added along the fitted function. The vertical black line represents results achieved with PBE0, i.e. with *α* = 0.25.

First, we consider the global hybrid functionals PBE0(*α*) and the dependence of the predicted band gaps on *α*. Figure 1 shows the band gaps as obtained with PBE (*α* = 0), PBE0 (*α* = 0.25), DD-PBE0 (*α* = 1/*ϵ*_*∞*_) and K-PBE0 (*α* = *α*_K_) as a function of the respective *α* values for all the materials considered in this work. As clearly seen in Fig. 1, the calculated band gaps closely follow a linear relationship with *α*. This linearity allows us to fit the band gap as a function of *α* and to find the fraction *α*_expt_ that reproduces the experimental band gap, thus providing a visual guidance for comparing the errors of each functional.

We first observe that the PBE band gaps are systematically smaller than the experimental ones, demonstrating the notorious band gap underestimation problem of semilocal functionals. As *α* increases, the band gaps become larger. The *α* values reproducing the experimental band gaps also tend to increase with increasing band gap. For PBE0, which includes a fixed Fock fraction of 0.25, the band gaps are overestimated in the small band gap regime and underestimated in the large band gap regime. A severe underestimation is observed for wide band gap materials such as Ar and LiF. This problem is greatly mitigated by adopting material-specific *α* values. Indeed, for both DD-PBE0 and K-PBE0, the respective *α* values fall much closer to *α*_expt_, yielding uniform accuracy over the whole band gap range. Between these two, K-PBE0 has a slight advantage over DD-PBE0 in terms of accuracy, producing a mean absolute error (MAE) of 0.34 eV compared to 0.41 eV for DD-PBE0 when compared for the same set of materials (see Supplementary Table 4).

This analysis of the role of *α* also sheds some light on the choice of *α*_*s*_ for RSH functionals. Going back to Eqs. (1) and (2), in the limit of *μ* → *∞*, the RSH functional falls back to PBE0(*α*_*l*_). In the limit of *μ* → 0, it falls back to PBE0(*α*_*s*_). In other words, tuning the value of *μ* essentially modulates the predicted band gap between PBE0(*α*_*l*_) and PBE0(*α*_*s*_). If we consider the class of range-separated functionals with *α*_*l*_ set to 1/*ϵ*_*∞*_ and *α*_*s*_ to 0.25, the tunable range of the predicted band gap is limited by the values from PBE0 and DD-PBE0. At variance, by setting *α*_*s*_ to 1, the tunable range is between the band gap values predicted by DD-PBE0 and PBE0(*α* = 1). Considering that PBE0(*α* = 1) largely overestimates the band gaps with respect to experimental values, selecting *α*_*s*_ = 1 yields a much larger tunable range of band gaps compared to that of *α*_*s*_ = 0.25. This observation helps us to better understand the influence of *μ* on the calculated band gaps for RSH functionals in the next section.

### Range-separated hybrid functionals

**Fig. 2:**
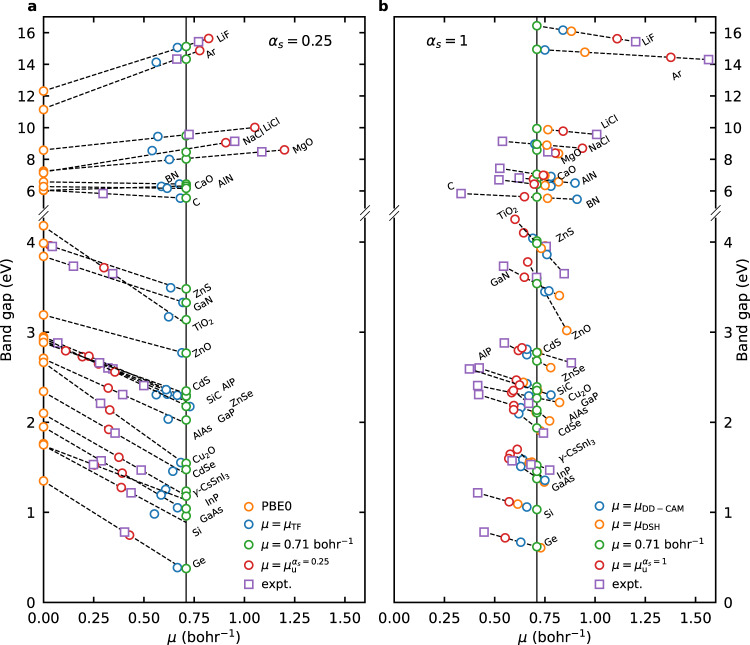
Band gap vs. *μ*. Band gaps as calculated by range-separated hybrid functionals with **a** *α*_*s*_ = 0.25 and **b** *α*_*s*_ = 1 as a function of *μ* for the 26 materials investigated, in comparison with experimental values. Data points for the same material are fitted to a linear relationship as indicated by the dashed lines. The experimental band gaps are then mapped to the fitted function. For ZnO, CaO, and BN in (**a**), the dependence of band gap on *μ* is so weak that such a mapping produces *μ* values beyond reasonable range and therefore the corresponding points are not shown. The vertical black line represents the results obtained with the fixed value of 0.71 bohr^−1^ for *μ*.

In this section, we examine how the choice of *μ* and *α*_*s*_ influence the accuracy of RSH functionals. Similar to the previous analysis for *α*, we show in Fig. 2 how the calculated band gaps depend on *μ* for the two classes of functionals with *α*_*s*_ = 0.25 and *α*_*s*_ = 1. Also in this case, we assume that *E*_*g*_ depends linearly on *μ* and find the *μ*_expt_ values that reproduce the experimental band gaps. The relationship can well be approximated as being linear (cf. Fig. 2).

We first look at how the changes in *μ* determine the band gaps. As has been established, varying *μ* tunes the predicted band gap between the values produced by PBE0(*α*_*l*_) and PBE0(*α*_*s*_). This leads to a major difference between the cases of *α*_*s*_ = 0.25 and *α*_*s*_ = 1. For *α*_*s*_ = 0.25 (Fig. 2a), the band gaps decrease with increasing *μ* for materials having *ϵ*_*∞*_ > 4 (mostly in the small band gap regime), and the reverse occurs for materials having *ϵ*_*∞*_ < 4 (mostly in the large band gap regime). This is because *α*_*s*_ is larger than *α*_*l*_ (1/*ϵ*_*∞*_) in the former group and *α*_*s*_ is smaller than *α*_*l*_ in the latter group. It also leads to the peculiar observation that for materials with *ϵ*_*∞*_ close to 4, changing *μ* has little effect on the predicted band gap, as manifested by the cases of CaO (*ϵ*_*∞*_ = 3.3), BN (*ϵ*_*∞*_ = 4.5), and ZnO (*ϵ*_*∞*_ = 3.74). For these materials, it is not possible to reproduce the experimental band gaps with reasonable values of *μ*. However, for *α*_*s*_ = 1 (Fig. 2b), it is ensured that *α*_*s*_ is larger than *α*_*l*_. As a result, the calculated band gaps always decrease with increasing *μ*. Generally, the selection of *α*_*s*_ = 1 creates a larger difference between *α*_*s*_ and *α*_*l*_ and thus a stronger dependence of the band gaps on *μ*.

With the general *E*_*g*_-vs-*μ* relationship established, we now take a closer look at the specific choices of *μ* values. When *α*_*s*_ is set to 0.25 (cf. Fig. 2a), we observe that the *μ*_TF_ values generally fall in the range of 0.6–0.8 bohr^−1^, close to the average *μ* value of 0.71 bohr^−1^. Consequently, the overall band gap accuracy of adopting *μ*_TF_ is almost the same as that of adopting the fixed value of 0.71 bohr^−1^. The MAEs of both functionals are 0.41 eV, and the mean absolute relative errors (MAREs) are 14.3% for the former and 14.6% for the latter, demonstrating little advantage of using material-specific *μ* values. The *μ* values reproducing experimental band gaps (henceforth referred to as *μ*_expt_) are in fact much more scattered. Considering functionals with *α*_*s*_ = 1 (cf. Fig. 2b), we find that DD-CAM and DSH perform better in terms of overall accuracy, producing MAEs of 0.23 and 0.24 eV, respectively (cf. Table 2). In Fig. 2b, we see that *μ*_DD-CAM_ and *μ*_DSH_ are also relatively close to the average value of 0.71 bohr^−1^. The MAE obtained with a fixed *μ* of 0.71 bohr^−1^ is 0.23 eV, again showing no advantage of using material-specific *μ* values.

**Table 2:** Band gaps (in eV) obtained with the hybrid functionals listed in Table 1 and the corresponding experimental references

	PBE0	DD-PBE0	HSE06	TF	${\mu }_{{{{\rm{fix}}}}}^{{\alpha }_{s} = 0.25}$	DSH	DD-CAM	${\mu }_{{{{\rm{fix}}}}}^{{\alpha }_{s} = 1}$	Expt.+ZPR
*sp* materials									
AlN	6.28	6.10	5.49	6.17	6.17	6.35	6.26	6.41	6.71
AlP	2.92	2.27	2.27	2.31	2.29	2.44	2.43	2.39	2.60
AlAs	2.71	2.01	1.93	2.04	2.03	2.11	2.16	2.11	2.31
Ar	11.14	14.62	10.36	14.13	14.33	14.77	14.91	14.95	14.33
BN	6.57	6.43	5.83	6.44	6.44	6.58	6.50	6.72	6.74^a^
C (diamond)	6.05	5.55	5.35	5.55	5.56	5.53	5.48	5.62	5.85^a^
CaO	6.03	6.33	5.30	6.29	6.30	6.97	6.92	7.06	7.43
LiCl	8.58	9.55	7.80	9.44	9.49	9.87	9.94	9.94	9.57
LiF	12.31	15.57	11.50	15.06	15.13	16.09	16.16	16.44	15.43
MgO	7.25	8.08	6.47	7.99	8.02	8.36	8.37	8.58	8.47
Si	1.76	0.95	1.14	0.98	0.96	1.09	1.06	1.03	1.22
SiC	2.91	2.30	2.24	2.31	2.30	2.35	2.30	2.35	2.59
NaCl	7.14	8.75	6.56	8.55	8.46	8.90	9.10	8.95	9.14
MAE_*sp*_	1.10	0.34	1.53	0.37	0.36	0.29	0.31	0.33	
MARE_*sp*_	15.9%	7.7%	18.3%	7.6%	7.7%	5.2%	5.6%	6.0%	
3*d* materials									
Ge	1.35	0.18	0.61	0.39	0.38	0.61	0.50	0.62	0.78
GaN	3.84	3.30	3.14	3.33	3.33	3.41	3.46	3.54	3.73
GaP	2.93	2.12	2.31	2.17	2.18	2.22	2.29	2.27	2.41
GaAs	1.95	0.95	1.26	1.05	1.04	1.34	1.51	1.37	1.57
InP	2.10	1.16	1.47	1.25	1.24	1.56	1.59	1.52	1.47
ZnO	3.19	2.72	2.43	2.77	2.77	3.02	3.45	3.60	3.61
ZnS	3.99	3.43	3.29	3.49	3.48	3.96	4.04	4.01	3.95
ZnSe	2.95	2.23	2.20	2.30	2.29	2.61	2.75	2.68	2.88
TiO_2_	4.18	3.01	3.39	3.17	3.14	3.93	3.86	3.99	3.65
Cu_2_O	2.66	1.52	1.89	1.55	1.54	2.02	2.49	2.13	2.21
CdS	2.89	2.37	2.19	2.36	2.35	2.77	2.95	2.77	2.66
CdSe	2.34	1.11	1.37	1.46	1.48	1.90	1.75	1.94	1.88
*γ*-CsSnI_3_	1.75	1.15	1.12	1.19	1.18	1.55	1.46^b^	1.46	1.53
MAE_*d*_	0.36	0.55	0.44	0.45	0.46	0.19	0.15	0.13	
MARE_*d*_	20.1%	26.7%	17.5%	21.0%	21.4%	8.4%	6.7%	6.3%	
MAE	0.73	0.44	0.98	0.41	0.41	0.24	0.23	0.23	
MARE	18.0%	17.2%	17.9%	14.3%	14.6%	6.8%	6.2%	6.1%	

The experimental band gaps are corrected for zero-point phonon renormalization (ZPR). The mean absolute errors (MAE) and mean absolute relative errors (MARE) with respect to the experimental references are calculated for the full set of materials and separately for the subgroups of *sp* and 3*d* materials.

^a^*GW* band gap from ref. ^58^.

^b^*μ*_DD−CAM_ taken as 0.71 bohr^−1^.

We now turn to the K-CAM functional in which *α*_*s*_ values are determined in a material-specific way by enforcing the generalized Koopmans’ condition. In this case, *μ* is fixed to 0.106 bohr^−1^, like in HSE06. When compared for the same set of materials, the K-CAM functional produces an MAE of 0.37 eV, which does not improve upon the MAE of 0.34 eV pertaining to the K-PBE0 functional (cf. Supplementary Table 4). This agrees with previous investigations adopting the same strategy for determining *α*_*s*_^37,39^. A recent study shows that it is possible to achieve a better accuracy by fixing *α*_*s*_ and determine *μ* through the enforcement of the generalized Koopmans’ condition^24^. However, we did not obtain such a higher accuracy when following an analogous strategy but with localized potential probes (see Supplementary information for more discussion).

### Optimizing the inverse screening parameter

With the insight into the *E*_*g*_-vs-*μ* relationship achieved above, we now inquire whether it is possible to devise a strategy for selecting *μ* that could further improve the accuracy of RSH functionals. In Fig. 2, we have seen that the TF, DD-CAM, and DSH functionals adopt *μ* values that fall close to the average value of 0.71 bohr^−1^, whereas the *μ*_expt_ values appear to be more scattered. We have also established that the dependence of *E*_g_ on *μ* is largely determined by the difference between *α*_*s*_ and *α*_*l*_. In particular, when *α*_*s*_ = *α*_*l*_, the change of *μ* has no effect on the calculated band gap. Prompted by this insight, we derive a relationship between *μ*_expt_, *α*_*s*_, and *α*_*l*_. Assuming that *E*_*g*_ depends linearly on *μ* as in Fig. 2, we have

5 $${E}_{g}(\mu )={E}_{g}({\mu }_{{{{\rm{expt}}}}})+(\mu -{\mu }_{{{{\rm{expt}}}}})\frac{d{E}_{g}(\mu )}{d\mu }.$$

**Fig. 3:**
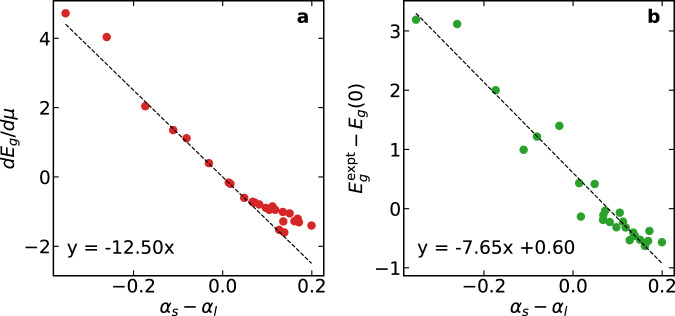
The linear dependence of *dE*_*g*_/*dμ* and $({E}_{g}^{{{{\rm{expt}}}}}-{E}_{g}(0))$ on (*α*_*s*_ − *α*_*l*_). Dependence of **a** *dE*_*g*_/*dμ* and **b** $({E}_{g}^{{{{\rm{expt}}}}}-{E}_{g}(0))$ on (*α*_*s*_ − *α*_*l*_) for the materials considered in this work in the case *α*_*s*_ = 0.25. Here, *E*_*g*_(0) represents the band gap obtained with *μ* = 0 and thus corresponds to the PBE0 band gap. The equations correspond to linear regressions of the calculated data points.

Considering that ${E}_{g}({\mu }_{{{{\rm{expt}}}}})={E}_{g}^{{{{\rm{expt}}}}}$, it follows that

6 $${\mu }_{{{{\rm{expt}}}}}=\frac{{E}_{g}^{{{{\rm{expt}}}}}-{E}_{g}(0)}{\frac{d{E}_{g}(\mu )}{d\mu }},$$

where *E*_*g*_(0) is the band gap value obtained with *μ* = 0, which coincides with the value obtained with PBE0(*α* = *α*_*s*_). Using the properties of the exchange potential, it can analytically be shown that *dE*_*g*_(*μ*)/*dμ* for a given material is proportional to *α*_*s*_ − *α*_*l*_ (cf. Section 6 in the Supplementary information). As seen in Fig. 3a, the proportionality constant is approximately constant for the materials considered in this work. To produce this figure, we set *α*_*s*_ = 0.25 and determine the derivative *dE*_*g*_(*μ*)/*dμ* by finite differences. Next, we focus on the numerator ${E}_{g}^{{{{\rm{expt}}}}}-{E}_{g}(0)$ in Eq. (6). From the success of DDH functionals, we can assume that ${E}_{g}^{{{{\rm{expt}}}}}\approx {E}_{g}[{{{\rm{PBE0}}}}(1/\epsilon )]$. Since *E*_*g*_(0) = *E*_*g*_[PBE0(*α*_*s*_)], we then infer that ${E}_{g}^{{{{\rm{expt}}}}}-{E}_{g}(0)$ relates to 1/*ϵ*_*∞*_ and thus to *α*_*l*_. In Fig. 3b, we show that this relationship can be closely approximated by a linear dependence of ${E}_{g}^{{{{\rm{expt}}}}}-{E}_{g}(0)$ on *α*_*s*_ − *α*_*l*_.

By combining the results from this analysis in Eq. (6), we propose a universal formula for selecting *μ* as a function of *α*_*s*_ and *α*_*l*_:

7 $${\mu }_{{{{\rm{u}}}}}=f\left(\frac{1}{{\alpha }_{l}-{\alpha }_{s}}\right),$$

where *f*(*x*) is a linear function. Based on this formula, we fit the *μ*_expt_ values obtained previously and arrive at the following expressions for *μ*_u_:

8 $${\mu }_{{{{\rm{u}}}}}^{{\alpha }_{s} = 0.25}=\frac{-0.044}{{\alpha }_{s}-{\alpha }_{l}}+0.65,$$

9 $${\mu }_{{{{\rm{u}}}}}^{{\alpha }_{s} = 1}=\frac{0.56}{{\alpha }_{s}-{\alpha }_{l}}-0.04.$$

**Fig. 4:**
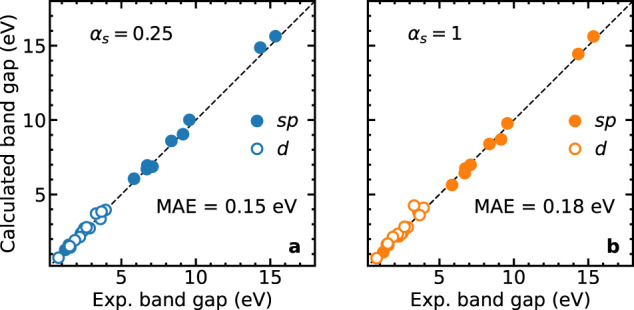
Band gaps obtained with *μ*_u_. Band gaps obtained with **a** ${\mu }_{{{{\rm{u}}}}}^{{\alpha }_{s} = 0.25}$ and **b** ${\mu }_{{{{\rm{u}}}}}^{{\alpha }_{s} = 1}$, compared to experimental band gaps. The disks and circles represent *sp* and 3*d* materials, respectively. The band gaps and mean absolute errors (MAEs) are given in Table 3.

The ${\mu }_{{{{\rm{u}}}}}^{{\alpha }_{s} = 0.25}$ and ${\mu }_{{{{\rm{u}}}}}^{{\alpha }_{s} = 1}$ values are shown on the respective panels of Fig. 2 and listed in Table 3. We observe that this expression correctly captures the divergence in the *E*_*g*_-vs-*μ* relationship at *α*_*s*_ = *α*_*l*_ and follows the scatter of *μ*_expt_. As such, these *μ*_u_ values further improve the accuracy in predicting band gaps compared to the previous functionals, with MAEs of 0.15 eV for ${\mu }_{{{{\rm{u}}}}}^{{\alpha }_{s} = 0.25}$ and of 0.18 eV for ${\mu }_{{{{\rm{u}}}}}^{{\alpha }_{s} = 1}$. Similarly, the respective MAREs reduce to 3.8% and 5.6%. The predicted band gaps are shown in Fig. 4 and are provided in Table 3. As shown in Fig. 4, the two functionals adopting *μ*_u_ values yield a uniform accuracy over the full range of band gaps. In the case *α*_*s*_ = 0.25, we remark that Eq. (8) leads to ${\mu }_{{{{\rm{u}}}}}^{{\alpha }_{s} = 0.25}$ values lying close to the divergence for materials with *ϵ*_*∞*_ ≈ 4. Nevertheless, the band gaps in these cases depend only very weakly on *μ*, and *μ* can thus be set to 0.

**Table 3:** The inverse screening parameters ${\mu }_{{{{\rm{u}}}}}^{{\alpha }_{s} = 0.25}$ and ${\mu }_{{{{\rm{u}}}}}^{{\alpha }_{s} = 1}$ (in bohr^−1^) and the corresponding band gaps (in eV)

	${\mu }_{{{{\rm{u}}}}}^{{\alpha }_{s} = 0.25}$	*E*_*g*_	${\mu }_{{{{\rm{u}}}}}^{{\alpha }_{s} = 1}$	*E*_*g*_	Expt. + ZPR
*sp* materials					
AlN	0.00	6.63	0.70	6.43	6.71
AlP	0.31	2.61	0.61	2.47	2.60
AlAs	0.35	2.35	0.60	2.19	2.31
Ar	0.74	14.71	1.38	14.44	14.33
BN	0.00	6.89	0.69	6.74	6.74
C(diamond)	0.06	6.00	0.65	5.64	5.85
CaO	1.88	6.78	0.74	6.99	7.43
LiCl	0.98	9.92	0.84	9.78	9.57
LiF	0.78	15.47	1.11	15.62	15.43
MgO	1.11	8.49	0.80	8.39	8.47
Si	0.41	1.25	0.57	1.12	1.22
SiC	0.24	2.69	0.62	2.41	2.59
NaCl	0.86	8.95	0.94	8.70	9.14
MAE_*sp*_		0.20		0.20	
MARE_*sp*_		3.1%		3.9%	
3*d* materials					
Ge	0.44	0.73	0.55	0.72	0.78
GaN	0.07	3.79	0.65	3.61	3.73
GaP	0.38	2.53	0.58	2.33	2.41
GaAs	0.41	1.41	0.57	1.60	1.57
InP	0.40	1.58	0.58	1.65	1.47
ZnO	0.00	3.29	0.66	3.78	3.61
ZnS	0.10	3.91	0.64	4.10	3.95
ZnSe	0.27	2.69	0.62	2.80	2.88
TiO_2_	0.33	3.67	0.60	4.25	3.65
Cu_2_O	0.36	2.09	0.59	2.35	2.21
CdS	0.17	2.75	0.64	2.83	2.66
CdSe	0.35	1.88	0.59	2.14	1.88
*γ*-CsSnI_3_	0.29	1.49	0.61	1.70	1.53
MAE_*d*_		0.10		0.17	
MARE_*d*_		4.4%		7.2%	
MAE		0.15		0.18	
MARE		3.8%		5.6%	

The mean absolute errors (MAE) and mean absolute relative errors (MARE) are calculated for the whole set, and for *sp* materials and *d* materials separately.

Furthermore, we test the proposed formulas for *μ* on several materials that are not part of the set studied. To verify that the proposed functionals with ${\mu }_{{{{\rm{u}}}}}^{{\alpha }_{s} = 0.25}$ and ${\mu }_{{{{\rm{u}}}}}^{{\alpha }_{s} = 1}$ do not spuriously open up a band gap for metallic systems, we consider graphite, sodium, and aluminum and observe no band gap opening for any of these metals. In addition, we remark that although the proposed *μ*_u_ formulas perform consistently on a large set of materials, there can be outliers for which DD functionals are less successful. For example, it has been shown in literature that DD hybrid functionals may lead to inaccurate band gaps in the case of correlated antiferromagnetic transition-metal oxides^52^. In the case of NiO, we indeed find that the functionals with ${\mu }_{{{{\rm{u}}}}}^{{\alpha }_{s} = 0.25}$ and ${\mu }_{{{{\rm{u}}}}}^{{\alpha }_{s} = 1}$ proposed here noticeably overestimate the band gap (see Supplementary information for more discussion).

### Conclusion

**Fig. 5:**
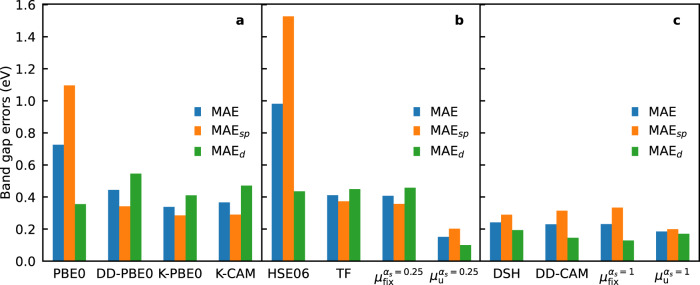
Errors of the functionals. Mean absolute errors (MAEs) of the predicted band gaps using **a** global functionals and piecewise linear hybrid functionals, **b** range-separated hybrid functionals with *α*_*s*_ = 0.25, and **c** range-separated hybrid functionals with *α*_*s*_ = 1.

To sum up, we have performed a comprehensive evaluation of the performance of available nonempirical hybrid functionals in predicting band gaps for a varied set of semiconductors and insulators. In Fig. 5, we provide a comparison of the MAEs for the functionals considered in this work. First, we have shown that the Fock fractions required for producing the experimental band gaps are material-specific. In most cases, they lie close to 1/*ϵ*_*∞*_. As a result, standard hybrid functionals such as PBE0 and HSE06 generally perform better for materials with medium band gaps, whereas they severely underestimate the band gaps of wide band gap materials. Adopting material-specific *α* values, as in DD-PBE0 and K-PBE0, greatly improves the uniformity of the accuracy over the band gap range. Between these two, K-PBE0 performs slightly better in terms of overall accuracy (MAE 0.34 eV compared to 0.44 eV). Going from K-PBE0 to K-CAM shows little improvement in the MAE. These three functionals also consistently show a better performance for *sp* materials compared to materials with 3*d* electrons.

As for the DD-RSH functionals, the class with *α*_*s*_ = 1 shows an overall advantage over the class with *α*_*s*_ = 0.25. The functional adopting *α*_*s*_ = 0.25 and *μ* = *μ*_TF_ produces an MAE of 0.41 eV, whereas DD-CAM and DSH have MAEs of 0.23 and 0.24 eV, respectively. It is also noteworthy that the accuracy of the latter group does not deteriorate as much for 3*d* materials compared to the global hybrid functionals, or to functionals with *α*_*s*_ = 0.25. In addition, we show that previous methods based on *μ*_TF_, *μ*_DSH_, and *μ*_DD-CAM_, all produce *μ* values fairly close to the average value of 0.71 bohr^−1^. In fact, functionals using a fixed *μ* of 0.71 bohr^−1^ are as accurate as methods adopting material-specific *μ*, consistent with previous findings by Chen et al.^20^.

Last, we demonstrate that a suitable choice of *μ* improves the accuracy of range-separated functionals even further. The *μ* values reproducing the experimental band gaps are far more scattered than any of the available schemes for determining *μ*. Based on this observation, we propose a new formula *μ*_u_, which correctly captures the divergence of *μ* at *α*_*s*_ = *α*_*l*_. This formula produces surprisingly good MAEs of 0.15 eV for *α*_*s*_ = 0.25 and 0.18 eV for *α*_*s*_ = 1, demonstrating the potential of further lowering the band gap errors achieved with RSH functionals. The RSH functionals constructed either with fixed *μ* values (0.71 bohr^−1^) or with *μ* values given by a simple analytical equation (*μ*_u_) provide a scheme that is much simplified with respect to the DD-CAM method^20^ or to the Koopmans construction process^23,37,38,41,43^. With these findings, we have established that hybrid functionals with material-specific parameters can approach the accuracy of state-of-the-art *GW* calculations with no greater computational cost than that of standard hybrid-functional calculations, making these functionals ideal candidates for widespread use in predicting electronic properties of solid-state materials.

## Methods

### Computational details

All DFT calculations are performed with the Quantum ESPRESSO suite^53^. Plane-wave basis sets for expanding the wave functions are used in conjunction with normconserving pseudopotentials including semicore *d* electrons^54,55^. The lattice parameters are taken from experimental values, as given in refs. ^20^ and ^50^. Plane-wave energy cut-offs and **k**-point grids are individually set for each material to ensure band gap convergence within 1 meV. Details of the material structures, convergence parameters, and specific *α* and *μ* values used for each functional can be found in the Supplementary information. For the DD-CAM functional, we take the *α*_*l*_ and *μ* values from ref. ^20^. For the K-PBE0 and K-CAM functional, *α*_K_ values are taken from ref. ^43^, but the accuracy of these schemes is here determined using the same experimental references as for the other functionals. The band gap calculations are repeated for all the functionals considered in this work to eliminate any effect resulting from the use of different pseudopotentials or materials structures.

The accuracy of the functionals considered in this work is determined with respect to experimental values corrected for zero-point renormalization. The sources of these values are given in Supplementary information. For BN and diamond, it is difficult to correct the measured optical band gaps for the excitonic effect, because these materials have indirect band gaps^56,57^. Thus, we use state-of-the-art *GW* calculations as a reference in these two cases^58^. Experimental errors still affect the accuracy determined for the various functionals, but the comparison between theory and experiment remains meaningful provided that the set of materials considered is large.

## Supplementary information

Supplementary Material

## Data Availability

The data associated with this work is available on Materials Cloud^59^.

## Code availability

The open-source Quantum ESPRESSO suite^53^ is freely available. The other relevant codes in this study are available from the corresponding authors upon reasonable request.
