# Supplementary material for: Range-separated hybrid functionals for accurate prediction of band gaps of extended systems
Source: NPJ Comput Mater. 2023 Jun 21;9(1):108. doi: 10.1038/s41524-023-01064-x (PMC11621024; doi:10.1038/s41524-023-01064-x)
Supplement: Supplementary file 1 — Supplementary Material [file 41524_2023_1064_MOESM1_ESM.pdf]

Supporting Information: Range-Separated Hybrid Functionals for Accurate Band Gap Prediction

Jing Yang[1*], Stefano Falletta[1] and Alfredo Pasquarello[1]

[1*]Chaire de Simulation à l'Echelle Atomique (CSEA), Ecole Polytechnique Fédérale de Lausanne (EPFL), Lausanne, CH-1015, Switzerland.

*Corresponding author(s). E-mail(s): jing.yang@epfl.ch;

1 Experimental band gaps and zero-point renormalization

Supplementary Table 1 Experimental band gap and zero-point renormalization (ZPR) values.

	Expt.	ZPR	Expt.+ZPR
AlN	6.23[1]	0.478[2]	6.71
AlP	2.52[1]	0.083[2]	2.60
AlAs	2.24[1]	0.067[2]	2.31
Ar	14.3 [3]	0.03[4]	14.33
BN	6.40[5]	0.016[2]	6.74[a]
C (diamond)	5.48[6]	0.026[2]	5.85[a]
CaO	7.09[7]	0.341[8]	7.43
LiCl	9.4[9]	0.17[10]	9.57
LiF	14.2[11]	1.23[8]	15.43
MgO	7.83[7]	0.64[2]	8.47
Si	1.17[12]	0.047[2]	1.22
SiC	2.42[13]	0.172[2]	2.59
NaCl	8.97[14]	0.17[10]	9.14
Ge	0.74[1]	0.042[2]	0.78
GaN	3.51[1]	0.223[2]	3.73
GaP	2.35[1]	0.057[2]	2.41
GaAs	1.52[1]	0.052[2]	1.57
InP	1.42[1]	0.05[15]	1.47
ZnO	3.44[16]	0.168[2]	3.61
ZnS	3.84[17]	0.112[2]	3.95
ZnSe	2.82[17]	0.061[2]	2.88
TiO_2	3.30[18]	0.349[8]	3.65
CdS	2.58[15]	0.08[2]	2.66
CdSe	1.85[15]	0.041[2]	1.88
Cu_2O	2.17[15]	0.03[15]	2.21
γ-$CsSnI_3$	1.25[19]	0.11[20]	1.53[b]

[a] The QS$G\tilde{W}^{\mathrm{NQ}}$ band gap from Ref. 21 is taken as reference in this case. [b] The correction in Ref. 20 includes both ZPR and spin-orbit coupling.

2 Materials structures and computational parameters

Supplementary Table 2 Materials structures and convergence parameters used for calculating the band gaps. The structures are taken from Refs. 22 and 20.

	$E_{\text{cutoff}}^{\text{DFT}}$ (Ry)	k-points	Space group	a_0 (Å)	c_0/a_0
AlN	85	8×8×8	$P6_3mc$	3.110	1.601
AlP	50	8×8×8	$F\bar{4}3m$	5.451	
AlAs	110	10×10×10	$F\bar{4}3m$	5.661	
Ar	60	8×8×8	$Fm\bar{3}m$	5.260	
BN	80	10×10×10	$F\bar{4}3m$	3.616	
C (diamond)	80	12×12×12	$Fd\bar{3}m$	3.567	
CaO	80	10×10×10	$Fm\bar{3}m$	4.803	
LiCl	90	8×8×8	$Fm\bar{3}m$	5.106	
LiF	80	8×8×8	$Fm\bar{3}m$	4.010	
MgO	80	8×8×8	$Fm\bar{3}m$	4.207	
Si	50	12×12×12	$Fd\bar{3}m$	5.430	
SiC	70	12×12×12	$F\bar{4}3m$	4.358	
NaCl	85	8×8×8	$Fm\bar{3}m$	5.595	
Ge	125	12×12×12	$Fd\bar{3}m$	5.658	
GaN	100	10×10×10	$P6_3mc$	3.189	1.626
GaP	110	10×10×10	$F\bar{4}3m$	5.448	
GaAs	110	10×10×10	$F\bar{4}3m$	5.648	
InP	90	12×12×12	$F\bar{4}3m$	5.866	
ZnO	90	10×10×10	$P6_3mc$	3.250	1.601
ZnS	80	10×10×10	$F\bar{4}3m$	5.410	
ZnSe	110	10×10×10	$F\bar{4}3m$	5.667	
TiO_2	90	8×8×8	$P4_2/mnm$	4.593	0.664
Cu_2O	90	10×10×10	$Pn\bar{3}m$	4.269	
CdS	100	10×10×10	$F\bar{4}3m$	5.818	
CdSe	120	10×10×10	$F\bar{4}3m$	6.052	
γ-$CsSnI_3$	100	4×4×4	$Pnma$	8.689	b = 8.638<br>c = 12.378

3 Dielectric constants and inverse screening parameters

Supplementary Table 3 The high-frequency dielectric constants ϵ_∞, the fractions of long-range Fock exchange $\alpha_l = 1/\epsilon_\infty$, and the inverse screening parameters (in bohr^{-1}) used for TF, DSH, and DD-CAM.

	ϵ_∞	α_l	μ_{TF}	μ_{DSH}	$\mu_{\mathrm{DD\text{-}CAM}}$
AlN	4.23	0.24	0.62	0.75	0.78
AlP	7.57	0.13	0.56	0.64	0.66
AlAs	8.70	0.11	0.62	0.71	0.63
Ar	1.66	0.60	0.56	0.95	0.75
BN	4.30	0.23	0.68	0.82	0.90
C (diamond)	5.47	0.18	0.84	0.80	0.91
CaO	3.56	0.28	0.59	0.75	0.78
LiCl	2.77	0.36	0.57	0.77	0.71
LiF	1.96	0.51	0.67	0.88	0.84
MgO	3.02	0.33	0.63	0.82	0.81
Si	12.24	0.08	0.55	0.61	0.66
SiC	6.54	0.15	0.62	0.71	0.78
NaCl	2.36	0.42	0.54	0.76	0.70
Ge	19.74	0.05	0.67	0.73	0.63
GaN	5.49	0.18	0.69	0.82	0.77
GaP	10.03	0.10	0.73	0.82	0.67
GaAs	12.69	0.08	0.67	0.75	0.63
InP	11.25	0.09	0.61	0.69	0.64
ZnO	4.96	0.20	0.69	0.86	0.75
ZnS	5.62	0.18	0.63	0.75	0.69
ZnSe	6.88	0.15	0.67	0.78	0.66
TiO_2	8.12	0.12	0.62	0.73	0.76
Cu_2O	8.95	0.11	0.68	0.77	0.71
CdS	5.96	0.17	0.61	0.71	0.66
CdSe	8.78	0.11	0.64	0.73	0.62
γ-$CsSnI_3$	7.24	0.14	0.59	0.67	0.75

4 K-PBE0 and K-CAM functionals

Supplementary Table 4 Band gaps calculated with K-PBE0 and K-CAM functionals and corresponding mixing parameters taken from Ref. 23. We recalculate the mean absolute error (MAE) with respect to the experimental references in Supplementary Table 1. Here, we also list the MAEs of the other functionals considered in this work calculated for the same nineteen materials.

	K-PBE0	α_{K}	K-CAM	$\alpha_{s,\mathrm{K}}$	Expt. + ZPR
AlP	2.31	0.14	2.31	0.15	2.60
AlAs	1.99	0.11	1.96	0.09	2.31
Ar	14.70	0.61	14.70	0.61	14.33
BN	6.53	0.24	6.79	0.26	6.44
C (diamond)	5.71	0.21	5.75	0.23	5.86
CaO	6.40	0.29	6.48	0.28	7.43
LiCl	9.65	0.37	9.64	0.37	9.57
LiF	15.83	0.53	15.76	0.52	15.43
MgO	8.32	0.34	8.35	0.35	8.47
Si	1.21	0.14	1.03	0.12	1.22
SiC	2.34	0.16	2.66	0.26	2.59
GaN	3.58	0.22	3.21	0.16	3.73
GaP	2.36	0.14	2.59	0.25	2.41
GaAs	1.00	0.09	1.02	0.09	1.57
InP	1.13	0.09	1.17	0.08	1.47
ZnO	3.13	0.25	3.22	0.25	3.61
ZnS	3.57	0.20	3.63	0.21	3.95
ZnSe	2.03	0.14	2.09	0.14	2.88
TiO_2	3.19	0.14	2.94	0.08	3.65
MAE	0.34		0.37		
	PBE0	DD-PBE0	HSE06	TF	$\mu_{\mathrm{fix}}^{\alpha_s=0.25}$
MAE	0.76	0.41	1.04	0.39	0.38
	DSH	DD-CAM	$\mu_{\mathrm{fix}}^{\alpha_s=1}$	$\mu_{\mathrm{u}}^{\alpha_s=0.25}$	$\mu_{\mathrm{u}}^{\alpha_s=1}$
MAE	0.27	0.25	0.27	0.19	0.17

5 Accuracy of K-CAM

In this work, we focus on a specific construction of the K-CAM functional, namely by fixing μ to 0.106 bohr^{-1} and α_s to $\alpha_{s,\mathrm{K}}$ obtained by enforcing the generalized Koopmans' condition on a localized potential probe [23]. As given in the main text, this type of K-CAM functional shows no improvement over the global K-PBE0 functional. However, adopting different construction schemes for the functional could lead to changes in the achieved accuracy. For instance, Wing et al. fixed α_s to 0.25 and μ to μ_K obtained by enforcing the generalized Koopmans' condition on localized Wannier functions. When this condition could not be satisfied at $\alpha_s = 0.25$, the value of α_s was gradually increased until a viable value of μ could be found. The resulting functional was found to yield an average error of 0.1 eV and a maximal error of 0.2 eV for the band gaps [24].

While the achieved accuracy is much appealing, we do not succeed in producing a K-CAM functional with a similar accuracy adopting the same strategy but with localized potential probes. One of the challenges here is the uncertainty in μ_K. To demonstrate this, we show in Supplementary Figure 1 the procedure for determining μ_K in LiF using two different finite-size correction schemes, the Freysoldt-Neugebauer-Van de Walle (FNV) scheme [25, 26] and the Makov-Payne (MP) scheme [27]. For permitting a consistent comparison, we use the same value of $\alpha_s = 0.25$ and the same $2\times2\times2$ supercell as in Ref. 24. From Supplementary Figure 1, we observe the following. First, the dependence of the single-particle energy levels of the occupied and unoccupied probe state on μ is weak, especially when the value of μ is large. This gives rise to uncertainties in determining the intersection point through interpolation. Second, the different finite-size correction schemes also lead to considerable differences in μ_K. In this case, the FNV and MP schemes give μ_K values of 0.62 and 0.76 bohr^{-1}, respectively, which correspond to band gaps of 15.14 and 15.37 eV. While both values are still satisfactory band gap predictions, discrepancies on this scale may affect the overall error with experiment. In Ref. 24, the MP scheme is used for the finite-size corrections during the construction of the functional. However, it is well-established that the FNV scheme produces improved results compared to the MP scheme [28].

We check whether the sensitivity of μ_K on the finite size corrections persists when higher values of α_s are used. For this purpose, we show in Supplementary Figure 2, the same procedure determining μ_K in the case of GaAs, for which $\alpha_s = 1$. We use the same $2\times2\times2$ supercell as in Ref. 24. We find that μ_K equals 1.08 and 0.69 bohr^{-1} when obtained with FNV and MP corrections, respectively. The respective band gaps are then 1.16 and 1.39 eV. Hence, the sensitivity of μ_K on the finite size corrections remains sizable also when $\alpha_s = 1$.

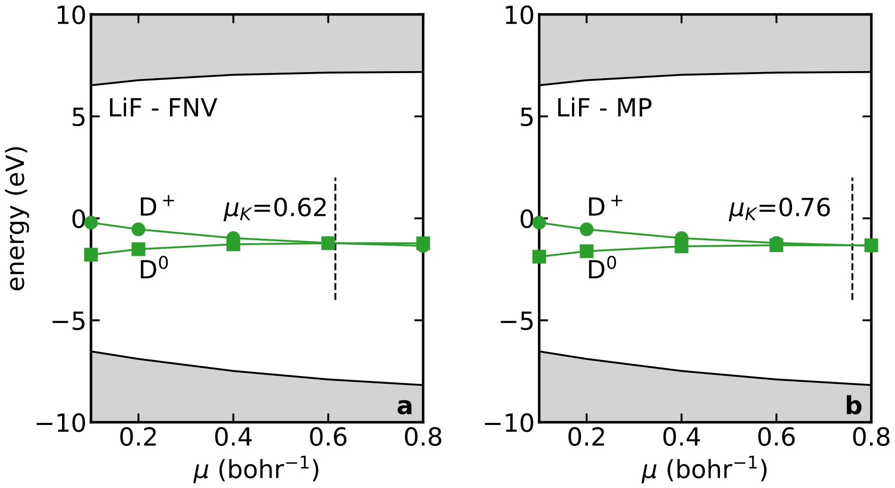

Supplementary Figure 1 Single particle energy levels of neutral and positively charged defect state (D^0 and D^+) in LiF at $\alpha_s = 0.25$ vs the inverse screening parameter μ, as calculated with (a) Freysoldt-Neugebauer-Van de Walle correction, and (b) Makov-Payne correction. The intersection point gives the determined μ_K satisfying the generalized Koopmans' condition. The localized defect state D is obtained through the use of a potential probe [23, 29].

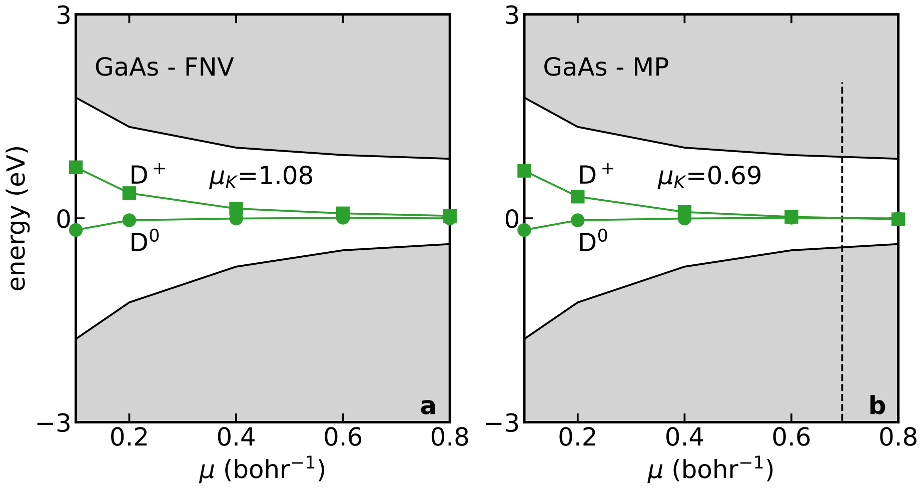

Supplementary Figure 2 Single particle energy levels of neutral and positively charged defect state (D^0 and D^+) in GaAs at $\alpha_s = 1$ vs the inverse screening parameter μ, as calculated with (a) Freysoldt-Neugebauer-Van de Walle correction, and (b) Makov-Payne correction. The intersection point gives the determined μ_K satisfying the generalized Koopmans' condition. The localized defect state D is obtained through the use of a potential probe [23, 29].

6 Dependence of $dE_g(\mu)/d\mu$ on $1/(\alpha_s - \alpha_l)$

In this section, we provide the derivation for the dependence of $dE_g(\mu)/d\mu$ on $1/(\alpha_s - \alpha_l)$. Using the Hellman-Feynman theorem and neglecting the variations of the wave functions with μ, we obtain

$$\frac{dE_g(\mu)}{d\mu} = \frac{d}{d\mu}(E_{\text{CBM}} - E_{\text{VBM}}) \tag{1}$$

$$= \langle\psi_{\text{CBM}}|\frac{dH}{d\mu}|\psi_{\text{CBM}}\rangle - \langle\psi_{\text{VBM}}|\frac{dH}{d\mu}|\psi_{\text{VBM}}\rangle, \tag{2}$$

where H represents the exchange-correlation Hamiltonian. For the range-separated hybrid functional, we have

$$H = \alpha_s v_x^{\text{Fock-SR}} + (1-\alpha_s)v_x^{\text{PBE-SR}} + \alpha_l v_x^{\text{Fock-LR}} + (1-\alpha_l)v_x^{\text{Fock-LR}}. \tag{3}$$

Consequently,

$$\begin{aligned}\langle\psi_{\text{CBM}}|\frac{dH}{d\mu}|\psi_{\text{CBM}}\rangle = {} & \alpha_s\langle\psi_{\text{CBM}}|\frac{dv_x^{\text{Fock-SR}}}{d\mu}|\psi_{\text{CBM}}\rangle + \alpha_l\langle\psi_{\text{CBM}}|\frac{dv_x^{\text{Fock-LR}}}{d\mu}|\psi_{\text{CBM}}\rangle \\ & +(1-\alpha_s)\langle\psi_{\text{CBM}}|\frac{dv_x^{\text{PBE-SR}}}{d\mu}|\psi_{\text{CBM}}\rangle \\ & +(1-\alpha_l)\langle\psi_{\text{CBM}}|\frac{dv_x^{\text{PBE-LR}}}{d\mu}|\psi_{\text{CBM}}\rangle\end{aligned} \tag{4}$$

We first focus on the first two terms on the right-hand side of Equation (4). We remark that v_x^{Fock} can be decomposed into a long-range and a short-range part:

$$v_x^{\text{Fock-SR}} = v_x^{\text{Fock}} \cdot \frac{1 - \text{erf}(\mu|\mathbf{r}-\mathbf{r}'|)}{|\mathbf{r}-\mathbf{r}'|} \tag{5}$$

$$v_x^{\text{Fock-LR}} = v_x^{\text{Fock}} \cdot \frac{\text{erf}(\mu|\mathbf{r}-\mathbf{r}'|)}{|\mathbf{r}-\mathbf{r}'|} \tag{6}$$

Therefore,

$$\begin{aligned}-\frac{dv_x^{\text{SR-Fock}}}{d\mu} &= \frac{dv_x^{\text{LR-Fock}}}{d\mu} \\ &= v_x^{\text{Fock}}\frac{d}{d\mu}\{\text{erf}(\mu|\mathbf{r}-\mathbf{r}'|)\}.\end{aligned} \tag{7}$$

Renaming $\langle\psi_{\text{CBM}}|v_x^{\text{Fock}} \cdot d\{\text{erf}(\mu|\mathbf{r}-\mathbf{r}'|)\}/d\mu|\psi_{\text{CBM}}\rangle = A$, the first two terms on the right-hand side of Equation (4) become $-A\alpha_s + A\alpha_l = -A(\alpha_s - \alpha_l)$. Similarly, taking $\langle\psi_{\text{CBM}}|v_x^{\text{PBE}} \cdot d\{\text{erf}(\mu|\mathbf{r}-\mathbf{r}'|)\}/d\mu|\psi_{\text{CBM}}\rangle = B$, the last two terms on the right-hand side of Equation (4) can be written as $-B(1-\alpha_s) + B(1-\alpha_l) = B(\alpha_s - \alpha_l)$. As such, we have proven that $\langle\psi_{\text{CBM}}|dH/d\mu|\psi_{\text{CBM}}\rangle$ is proportional to $\alpha_s - \alpha_l$. Following the

same derivation, $\langle\psi_{\mathrm{VBM}}|dH/d\mu|\psi_{\mathrm{VBM}}\rangle$ is also proportional to $\alpha_s - \alpha_l$. Going back to Equation (2), we arrive at

$$\frac{dE_g(\mu)}{d\mu} = C(\alpha_s - \alpha_l), \tag{8}$$

where C is a proportionality constant independent of α_s and α_l.

7 Additional information on the errors found with the various functionals

Supplementary Table 5 Mean error (ME), mean relative error (MRE), mean absolute error (MAE), mean absolute relative error (MARE), maximum absolute error ($\mathrm{AE}^{\mathrm{max}}$), maximum absolute relative error ($\mathrm{ARE}^{\mathrm{max}}$) of the functionals in Table 1 of the main text. The errors are calculated for the full set of materials and separately for the subgroups of *sp* and *d* materials. The errors are given in units of eV.

	PBE0	DD-PBE0	HSE06	TF	$\mu_{\mathrm{fix}}^{\alpha_s=0.25}$	DSH	DD-CAM	$\mu_{\mathrm{fix}}^{\alpha_s=1}$	$\mu_{\mathrm{u}}^{\alpha_s=0.25}$	$\mu_{\mathrm{u}}^{\alpha_s=1}$
ME_{sp}	0.80	0.28	1.53	0.37	0.36	0.05	0.05	−0.04	−0.10	0.12
ME_{d}	−0.29	0.55	0.44	0.45	0.46	0.11	0.02	0.03	−0.00	−0.12
ME	0.26	0.41	0.98	0.41	0.41	0.08	0.04	−0.00	−0.05	−0.00
MRE_{sp}	1.7%	7.3%	18%	7.6%	7.7%	3.3%	3.6%	2.8%	−1.8%	3.2%
MRE_{d}	−18%	27%	18%	21%	21%	5.2%	1.6%	2.9%	0.06%	−4.49%
MRE	−8.3%	17%	18%	14%	15%	4.3%	2.6%	2.9%	−0.86%	−0.65%
MAE_{sp}	1.10	0.34	1.53	0.37	0.36	0.29	0.31	0.33	0.20	0.20
MAE_{d}	0.36	0.55	0.44	0.45	0.46	0.19	0.15	0.13	0.10	0.17
MAE	0.73	0.44	0.98	0.41	0.41	0.24	0.23	0.23	0.15	0.18
MARE_{sp}	16%	7.7%	18.3%	7.6%	7.7%	5.2%	5.6%	6.0%	3.1%	3.9%
MARE_{d}	20%	27%	18%	21%	21%	8.4%	6.7%	6.3%	4.4%	7.2%
MARE	18%	17%	18%	14%	15%	6.8%	6.2%	6.1%	3.8%	5.6%
$\mathrm{AE}_{sp}^{\mathrm{max}}$	3.19	1.10	3.97	1.14	1.13	0.66	0.73	1.01	0.56	0.44
$\mathrm{AE}_{d}^{\mathrm{max}}$	0.63	0.89	1.18	0.84	0.84	0.59	0.27	0.34	0.25	0.60
$\mathrm{AE}^{\mathrm{max}}$	3.19	1.10	3.97	1.14	1.13	0.66	0.73	1.01	0.56	0.60
$\mathrm{ARE}_{sp}^{\mathrm{max}}$	45%	22%	29%	19%	21%	10%	13%	15%	7.6%	8.2%
$\mathrm{ARE}_{d}^{\mathrm{max}}$	73%	77%	33%	51%	52%	22%	14%	21%	9.6%	17%
$\mathrm{ARE}^{\mathrm{max}}$	73%	77%	33%	51%	52%	22%	14%	21%	10%	17%

8 The case of correlated oxides

In the case of NiO, Chen *et al.* (Ref. 22) and Liu *et al.* (Ref. 30) found largely differing band gaps, while apparently applying the same self-consistent DD-RSH-CAM scheme (cf. Supplementary Table 6). More generally, Liu *et al.* reported that self-consistent DD-CAM functionals systematically overestimate the band gaps of correlated antiferromagnetic transition-metal oxides, namely FeO, MnO, CoO, and NiO [30]. They also

found that DD-CAM and DSH functionals significantly underestimate the dielectric constants of these materials. Here, we test the proposed functionals with $\mu_{\mathrm{u}}^{\alpha_s=0.25}$ and $\mu_{\mathrm{u}}^{\alpha_s=1}$ on the problematic case of NiO and compare our results with those of Refs. 22 and 30 (see Supplementary Table 6). In our functionals, we use the experimentally measured value of the dielectric constant, which corresponds to 5.7. Both functionals yield overestimated band gaps, thereby confirming the tendency observed by Liu *et al.* Hence, our results also suggest that dielectric-dependent hybrid functionals may be subject to inaccuracies when applied to correlated transition-metal oxides, possibly due to their correlated character.

Supplementary Table 6 The band gap of NiO calculated with DD-CAM in Refs. 22 and 30, and with $\mu_{\mathrm{u}}^{\alpha_s=0.25}$ and $\mu_{\mathrm{u}}^{\alpha_s=1}$ proposed in this work. The ϵ_∞, α_l, and μ values used are also listed. Here, the experimental band gap is not corrected for ZPR.

	ϵ_∞	α_l	μ	E_g	$\epsilon_\infty^{\mathrm{expt}}$	E_g^{expt}
Chen *et al.* (Ref. 22)	7.16	0.14	0.82	4.68	5.7	4.3
Liu *et al.* (Ref. 30)	3.57	0.28	0.83	6.34		
$\mu_{\mathrm{u}}^{\alpha_s=0.25}$	5.7	0.18	0.06	5.15		
$\mu_{\mathrm{u}}^{\alpha_s=1}$	5.7	0.18	0.64	6.18		

References

[1] Vurgaftman, I., Meyer, J. R. & Ram-Mohan, L. R. Band parameters for iii–v compound semiconductors and their alloys. *Journal of Applied Physics* **89**, 5815–5875 (2001).

[2] Brousseau-Couture, V., Godbout, E., Côté, M. & Gonze, X. Zero-point lattice expansion and band gap renormalization: Grüneisen approach versus free energy minimization. *Phys. Rev. B* **106**, 085137 (2022).

[3] Baldini, G. Ultraviolet absorption of solid argon, krypton, and xenon. *Phys. Rev.* **128**, 1562–1567 (1962).

[4] Tal, A., Liu, P., Kresse, G. & Pasquarello, A. Accurate optical spectra through time-dependent density functional theory based on screening-dependent hybrid functionals. *Phys. Rev. Res.* **2**, 032019 (2020).

[5] Chrenko, R. Ultraviolet and infrared spectra of cubic boron nitride. *Solid State Communications* **14**, 511–515 (1974).

[6] Clark, C. D., Dean, P. J., Harris, P. V. & Price, W. C. Intrinsic edge absorption in diamond. *Proc. Math. Phys. Eng. Sci. P ROY SOC A-MATH PHY* **277**, 312–329 (1964).

[7] Whited, R., Flaten, C. J. & Walker, W. Exciton thermoreflectance of mgo and cao. *Solid State Communications* **13**, 1903–1905 (1973).

[8] Engel, M. *et al.* Zero-point renormalization of the band gap of semiconductors and insulators using the projector augmented wave method. *Phys. Rev. B* **106**, 094316 (2022).

[9] Baldini, G. & Bosacchi, B. Optical properties of na and li halide crystals at 55 °k. *physica status solidi (b)* **38**, 325–334 (1970).

[10] Lambrecht, W. R. L., Bhandari, C. & van Schilfgaarde, M. Lattice polarization effects on the screened coulomb interaction w of the gw approximation. *Phys. Rev. Mater.* **1**, 043802 (2017).

[11] Piacentini, M., Lynch, D. W. & Olson, C. G. Thermoreflectance of lif between 12 and 30 ev. *Phys. Rev. B* **13**, 5530–5543 (1976).

[12] Bludau, W., Onton, A. & Heinke, W. Temperature dependence of the band gap of silicon. *Journal of Applied Physics* **45**, 1846–1848 (1974).

[13] Humphreys, R., Bimberg, D. & Choyke, W. Wavelength modulated absorption in sic. *Solid State Communications* **39**, 163–167 (1981).

[14] Roessler, D. M. & Walker, W. C. Electronic spectra of crystalline nacl and kcl. *Phys. Rev.* **166**, 599–606 (1968).

[15] Cardona, M. & Thewalt, M. L. W. Isotope effects on the optical spectra of semiconductors. *Rev. Mod. Phys.* **77**, 1173–1224 (2005).

[16] Mang, A., Reimann, K. & Rübenacke, S. Band gaps, crystal-field splitting, spin-orbit coupling, and exciton binding energies in zno under hydrostatic pressure. *Solid State Communications* **94**, 251–254 (1995).

[17] Mang, A., Reimann, K., Rübenacke, S. & Steube, M. Two-photon spectroscopy study of zns and cds under hydrostatic pressure. *Phys. Rev. B* **53**, 16283–16288 (1996).

[18] Tezuka, Y. *et al.* Photoemission and bremsstrahlung isochromat spectroscopy studies of tio2 (rutile) and srtio3. *Journal of the Physical Society of Japan* **63**, 347–357 (1994).

[19] Tao, S. *et al.* Absolute energy level positions in tin- and lead-based halide perovskites. *Nature Communications* **10**, 2560 (2019).

[20] Wang, H., Tal, A., Bischoff, T., Gono, P. & Pasquarello, A. Accurate and efficient band-gap predictions for metal halide perovskites at finite temperature. *npj Comput. Mater.* **8**, 237 (2022).

[21] Tal, A., Chen, W. & Pasquarello, A. Vertex Function Compliant with the Ward Identity for Quasiparticle Self-Consistent Calculations Beyond GW. *Phys. Rev. B* **103**, L161104 (2021).

[22] Chen, W., Miceli, G., Rignanese, G.-M. & Pasquarello, A. Nonempirical dielectric-dependent hybrid functional with range separation for semiconductors and insulators. *Phys. Rev. Mater.* **2**, 073803 (2018).

[23] Yang, J., Falletta, S. & Pasquarello, A. One-shot approach for enforcing piecewise linearity on hybrid functionals: Application to band gap predictions. *J. Phys. Chem. Lett.* **13**, 3066–3071 (2022).

[24] Wing, D. *et al.* Band gaps of crystalline solids from wannier-localization–based optimal tuning of a screened range-separated hybrid functional. *Proc. Natl. Acad. Sci.* **118** (2021).

[25] Freysoldt, C., Neugebauer, J. & Van de Walle, C. G. Fully ab initio finite-size corrections for charged-defect supercell calculations. *Phys. Rev. Lett.* **102**, 016402 (2009).

[26] Chen, W. & Pasquarello, A. Correspondence of defect energy levels in hybrid density functional theory and many-body perturbation theory. *Phys. Rev. B* **88**, 115104 (2013).

[27] Makov, G. & Payne, M. C. Periodic boundary conditions in ab initio calculations. *Phys. Rev. B* **51**, 4014–4022 (1995).

[28] Komsa, H.-P., Rantala, T. T. & Pasquarello, A. Finite-size supercell correction schemes for charged defect calculations. *Phys. Rev. B* **86**, 045112 (2012).

[29] Bischoff, T., Reshetnyak, I. & Pasquarello, A. Adjustable potential probes for band-gap predictions of extended systems through nonempirical hybrid functionals. *Phys. Rev. B* **99**, 201114 (2019).

[30] Liu, P., Franchini, C., Marsman, M. & Kresse, G. Assessing model-dielectric-dependent hybrid functionals on the antiferromagnetic transition-metal monoxides mno, feo, coo, and nio. *J. Phys. Condens.* **32**, 015502 (2019).
